# Geotechnical data compilation for evaporitic rocks in Abu Dhabi, UAE: A resource for engineers

**DOI:** 10.1016/j.dib.2024.110322

**Published:** 2024-03-16

**Authors:** Hasan Arman, Ahmed Gad, Osman Abdelghany, Bahaa Mahmoud, Ala Aldahan, Safwan Paramban, Mahmoud Abu Saima

**Affiliations:** a Geosciences Department, College of Science, United Arab Emirates University, Al Ain 15551, United Arab Emirates; b Geology Department, Faculty of Science, Ain Shams University, Cairo 11566, Egypt

**Keywords:** Evaporites, Geoengineering properties, Degradability, Rock strength, Foundation instability, Sustainable development

## Abstract

The durability and degradability of evaporitic rocks are always a critical concern in geological, civil, and geotechnical engineering applications, necessitating careful consideration for reliable, secure, and sustainable construction. This dataset is based on a comprehensive collection of geotechnical data involving both mechanical and physical properties as well as geochemical analyses for the evaporitic rocks in Abu Dhabi and its vicinity. The purpose of this dataset is to be an important source for construction professionals, engineers, and decision-makers in the region by providing basic insights into the challenges associated with building projects on and in evaporitic rocks. This data is obtained from extensive field investigations and laboratory analyses and will help to ensure that construction activities are carried out safely and efficiently when dealing with evaporitic rock formations. The prevalence of evaporitic rocks in Abu Dhabi highlights the importance of this geotechnical data compilation in facilitating informed decision-making and supporting safe construction.

Specifications Table

**Table:** 

Subject	Geotechnical Engineering and Engineering Geology.
Specific subject area	Mechanical and physical properties and geochemical composition of evaporitic Rocks
Type of data	Table
Data collection	The studied evaporitic rock blocks were collected during extensive field investigations in Abu Dhabi and its vicinity, UAE. Laboratory tests of mechanical (Uniaxial Compressive Strength, Point Load Index, Indirect Tensile Strength, Schmidt Hardness Value, Sonic Wave Velocity, and Slake Durability Index) and physical (Density, Unit Weight, Specific Gravity, Water Content, Porosity, and Void Ratio) were conducted on the rock blocks and cores with specified dimensions, following procedures and utilizing equipment exclusively adopted in international standard methods (further information is included in the Experimental Design, Materials, and Methods section).
Data source location	Region: Middle East Country: United Arab Emirates City: Abu Dhabi (24° 0ʹ - 24° 40ʹ N and 54° 0ʹ - 55° 0ʹ E) The studied evaporitic rock blocks were collected from Abu Dhabi and its vicinity. The coordinates and location of each sampling site are included in Fig. 1 and Table 1. The samples are stored and analyzed at the Geosciences Department, College of Science, United Arab Emirates University, Al Ain 15551, United Arab Emirates.
Data accessibility	Repository name: Comprehensive Analysis of Mechanical, Physical, and Geochemical Properties of Evaporitic Rocks in Abu Dhabi, UAE. [1] Data identification number: 10.17632/z7t3c2dmpz.1 Direct URL to data: https://data.mendeley.com/datasets/z7t3c2dmpz/1

## 1 Value of the Data

• In Abu Dhabi, a rapidly developing region with extensive infrastructure projects where evaporitic rocks are prevalent, their study provides a significant contribution to the region. The unique challenges posed by evaporitic rocks in Abu Dhabi, such as their easily degradable nature leading to surface subsidence, sinkhole formation, and landslides. Construction businesses, engineers, and decision-makers can benefit greatly from the insights gained, particularly in addressing foundation issues at construction sites.
• Raw datasets obtained from extensive field investigations and laboratory analyses are presented on geotechnical (mechanical and physical) properties and geochemical composition measured in evaporitic rocks from Abu Dhabi and its vicinity, UAE. This data will help to ensure that construction activities are carried out safely and efficiently when dealing with evaporitic rock formations.
• The geotechnical data presented in this dataset will be essential tools for navigating the difficulties of construction in Abu Dhabi and its vicinity.
• This geotechnical data compilation for evaporitic rocks emerges not only as a scholarly endeavor but as a feasible resource for informed decision-making in the engineering and construction sectors in this region.
• As urban areas expand, this compilation is a valuable resource for construction businesses, engineers, and decision-makers in Abu Dhabi, providing essential information for safe and effective construction practices in the presence of evaporitic rocks.
• The presented dataset can be freely and easily used by interested readers. The geochemical data can potentially be correlated with geotechnical parameters. This correlation can be utilized to predict certain geotechnical properties of evaporitic rocks, such as strength, durability, and rock-water interaction, which are crucial for engineering design.

## 2 Background

In arid regions, the investigation of evaporitic rocks provides useful information due to their complexity and diverse geological, environmental, and geotechnical contributions [2], [3], [4], [5], [6]. This distinction points out the need for a comprehensive study that not only addresses origin and paleoenvironmental conditions but also addresses engineering issues, particularly in construction [6], [7], [8].

The importance of understanding the mechanical and physical properties of evaporitic rocks is important for engineers and construction professionals [9,10]. Geotechnical data facilitates quantitative analysis, providing insights into long-term behavior, stability, and resource management [11,12]. These implications extend beyond the immediate regions, resulting in a better understanding of evaporite sequences worldwide [13]. The examination of geotechnical data is a key factor in determining the durability of subsurface evaporitic rocks and underground structures. This knowledge provides the basis for comprehensive risk assessment and early risk mitigation [2,[14], [15], [16]]. Furthermore, examining the geotechnical aspects of evaporitic rocks sheds light on their stability over geological time, aqueous geochemistry, water-rock interactions, and stress redistribution during excavation [17], [18], [19]. These aspects are important to understand the geotechnical properties of the surrounding rocks, thus influencing construction practices.

## 3 Data Description

The dataset [1] encompasses a wide array of mechanical properties, including Uniaxial Compressive Strength (UCS), Point Load Index (PLI), Indirect Tensile Strength (ITS), Schmidt Hardness Value (SHV), Sonic Wave Velocity (SWV), and Slake Durability Index (SDI). These properties provide essential insights into the strength, durability, and deformation characteristics of the evaporitic rocks, forming the basis for engineering design and risk assessment. In addition to the mechanical properties, the dataset examines key physical attributes, such as Density (ρ), Unit Weight (γ), Specific Gravity (G_s_), Water Content (W_c_), Porosity (n), and Void Ratio (e). These parameters offer a comprehensive understanding of the rock's mass, volume, and water-related characteristics, contributing to the assessment of resistance, permeability, and overall geological stability. These data were modeled and validated using HYFIS vs FMR, LWR, and Least squares regression methods [20]. Furthermore, the geochemical composition (major elements) of the investigated evaporitic rocks is presented. The geochemical composition of these rocks provides important information about their chemical characteristics, which is required to assess their suitability for various geotechnical applications by predicting any potential reactions or alterations to determine their environmental stability under specific conditions.

The data obtained from each analysis are presented and described in dataset Tables 1–3 [1]. The dataset directory structure is as follows:

- Table 1.xlsx: A file representing the mechanical parameters test results of evaporitic rocks, Abu Dhabi.
- Table 2. xlsx: A file representing the physical parameters test results of evaporitic rocks, Abu Dhabi.
- Table 3. xlsx: A file representing the concentration of major elements (oxides%) of evaporitic rocks, Abu Dhabi.

## 4 Experimental Design, Materials and Methods

### 4.1 The study area and field sampling

The land surface of Abu Dhabi and its vicinity is predominantly composed of a 1–2 m thick gypcrete soil derived from recent eolian. The soil is covered by approximately 2 m of dune sand. The gypcrete soils are underlined by a sequence of Lower Miocene evaporitic rocks (18–20 m thick) [6,8].

**Fig. 1:**
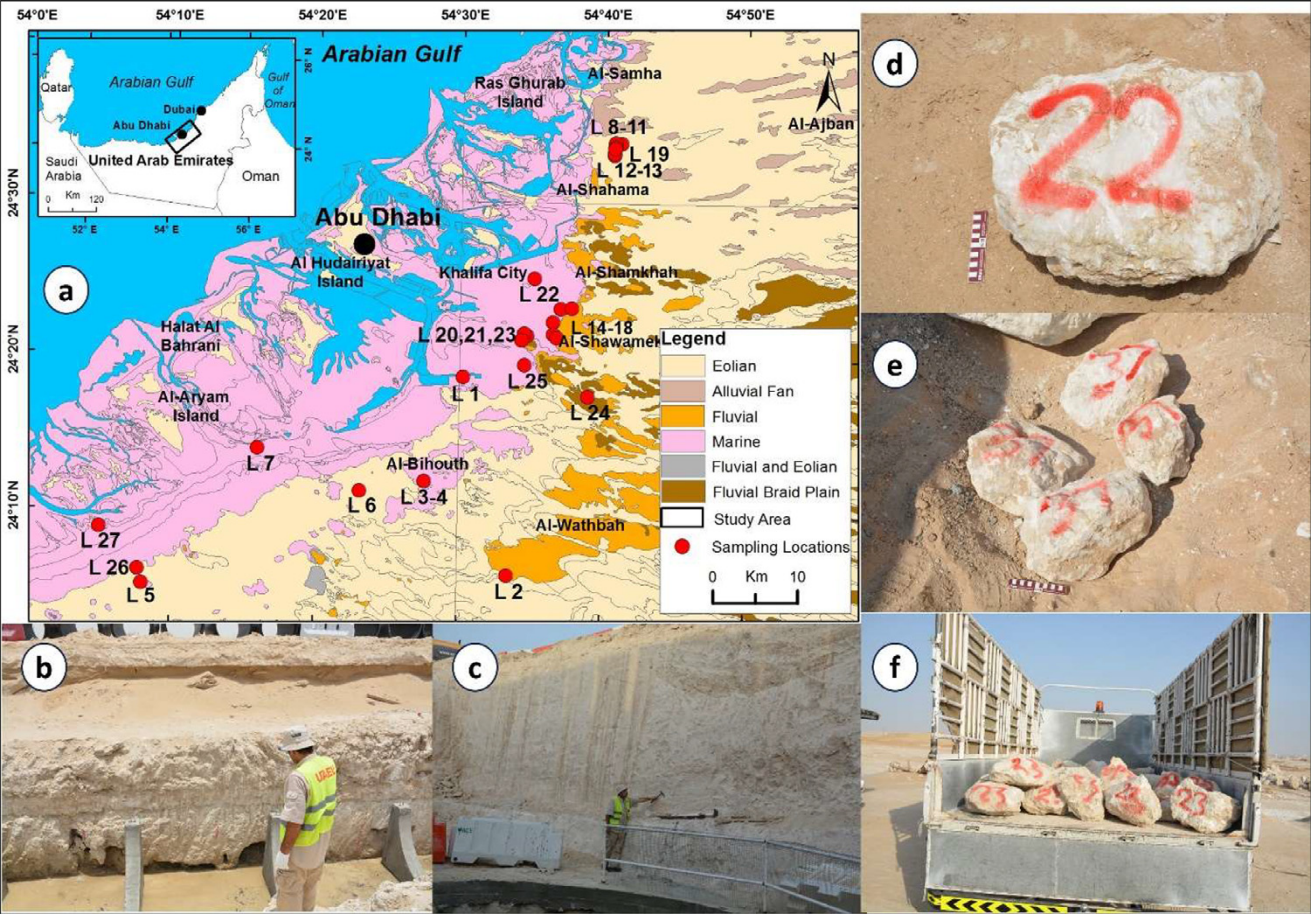
(a) Geological map of Abu Dhabi and sampling locations; (b) and (c) temporary excavations and boreholes; (d) and (e) rock block samples; (f) transportation of rock block samples to the laboratory.

Detailed field investigations of evaporite rocks were conducted at twenty-seven different locations in Abu Dhabi (L1-L27) (Fig. 1a and Table 1). 149 representative evaporitic rock blocks were collected from temporary excavations and boreholes that reached depths of up to 20 m (Fig. 1b, c). Evaporitic rock blocks were carefully inspected and those without visible defects such as cracks, fractures or alteration zones were transported to a laboratory and stored under laboratory conditions (Fig. 1d-f).

**Table 1:** Sampling sites and the tested rock blocks from each site.

Sampling sites	Coordinates		Tested Rock Blocks
	Long.	Lat.	
L1	E 54° 30.255′	N 24° 18.767′	1
L2	E 54° 28.777′	N 24° 14.580′	6
L3	E 54° 27.659′	N 24° 12.076′	1
L4	E 54° 27.659′	N 24° 12.076′	1
L5	E 54° 07.994′	N 24° 05.272′	6
L6	E 54° 23.128′	N 24° 11.369′	3
L7	E 54° 15.979′	N 24° 14.033′	1
L8	E 54° 41.116′	N 24° 33.829′	40
L9	E 54° 40.622′	N 24° 33.087	14
L10	E 54° 40.624′	N 24° 33.103′	9
L11	E 54° 40.666′	N 24° 33.521′	11
L12	E 54° 40.684′	N 24° 33.614′	2
L13	E 54° 40. 675′	N 24° 33. 545′	3
L14	E 54° 36.737′	N 24° 21.338′	3
L15	E 54° 36.508′	N 24° 21.552′	10
L16	E 54° 36.490′	N 24° 22.347′	2
L17	E 54° 37.785′	N 24° 23.252′	1
L18	E 54° 37.010′	N 24° 23.185′	1
L19	E 54° 40.706′	N 24° 33.848′	4
L20	E 54° 34.302′	N 24° 21.176′	3
L21	E 54° 34.495′	N 24° 21.591′	3
L22	E 54° 35.053′	N 24° 25.128′	5
L23	E 54° 35.020′	N 24° 21.417′	7
L24	E 54° 38.969′	N 24° 17.606′	4
L25	E 54° 34.518′	N 24° 19.585′	4
L26	E 54° 07.710′	N 24° 06.185′	2
L27	E 54° 04.979′	N 24° 08.870′	2
Total			149

### 4.2 Experiments

**Fig. 2:**
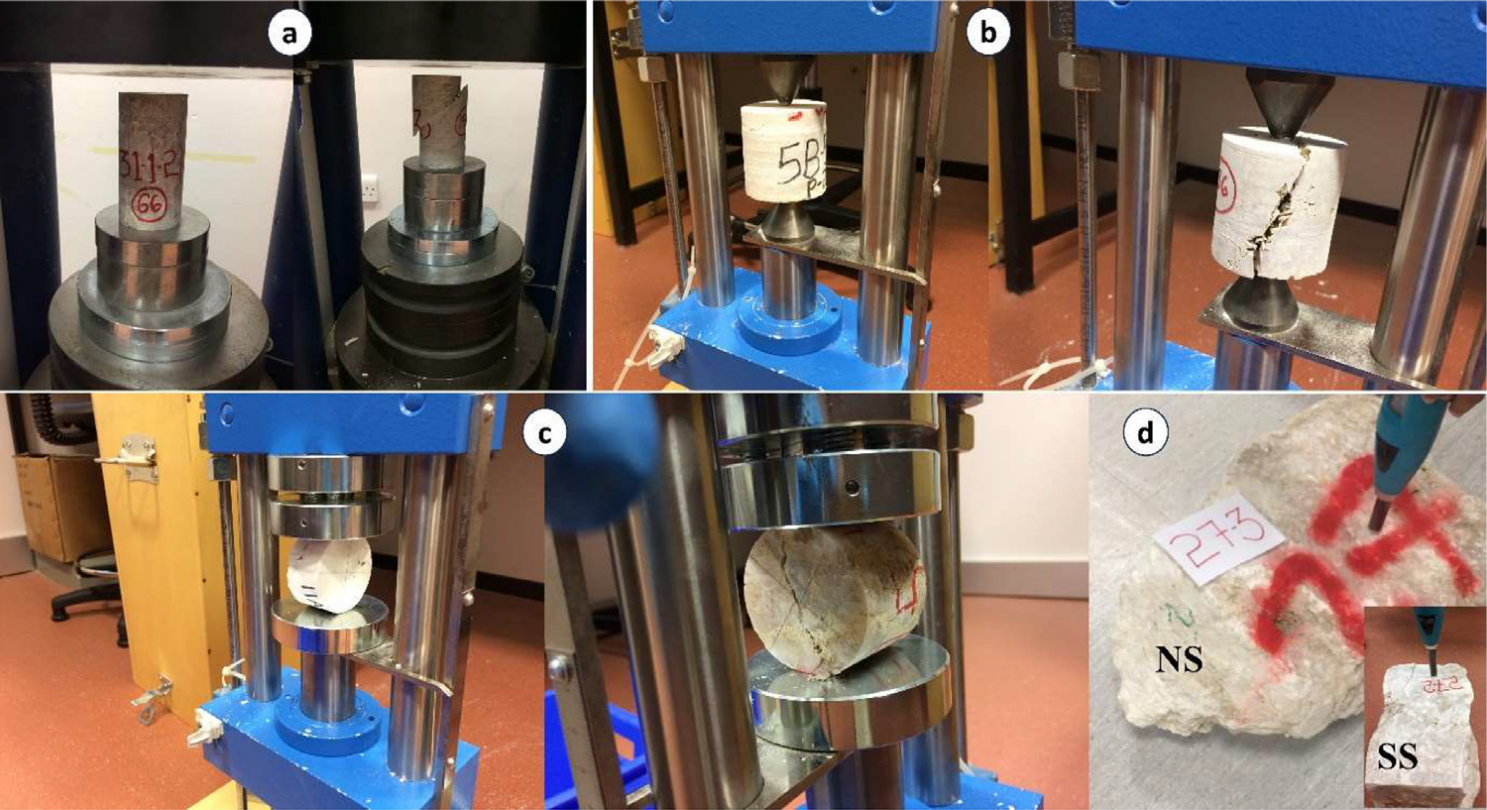
(a) Uniaxial Compressive Strength (UCS) test on the core sample; (b) Point Load Index (PLI) test on the core sample; (c) Indirect Tensile Strength (ITS) test on the core sample; (d) Schmidt Hammer test on the rock block sample (SHV_RB_) with a natural surface (NS) and saw-cut surface (SS).

**Fig. 3:**
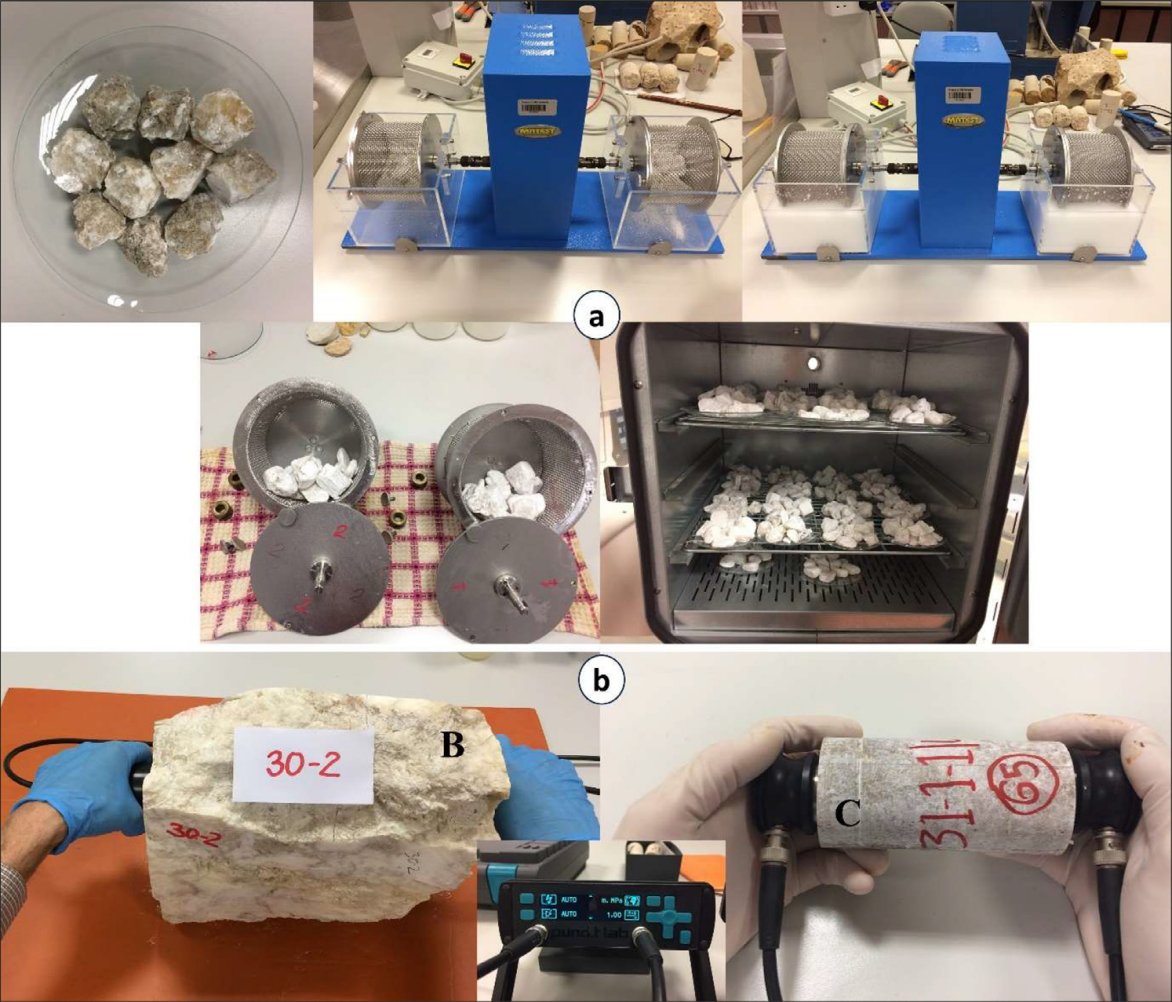
(a) Slake Durability Index (SDI) test on rock lumps; (b) Sonic Wave Velocity (SWV) test on block (B) and core (C) samples.

Evaporite rock blocks without major macroflaws were transported to the laboratory for the preparation of NX-size cores (54 mm). Following the coring process, core samples were trimmed on both sides, considering the available core length and maintaining a length-to-diameter ratio of approximately 2:1 for subsequent geotechnical measurements. It was ensured that both sides of each sample were identical within the specified limits. Before testing, the diameter and length of each sample were measured, the samples were weighed, and all relevant data was thoroughly recorded in a datasheet. A considerable number of samples were tested in accordance with relevant standards [21], [22], [23], [24], [25], [26], [27], [28] to determine the mechanical and physical properties of the collected evaporitic rocks (Table 2 and Figs. 2 and 3). During the SHR_RB_ measurements, maximum precautions were taken to avoid any influence from obvious fractures, discontinuities, or proximity to edges, as these factors could influence the results. The SDI tests were conducted applying both distilled water and sea water as the slaking fluid and classified based on Franklin and Chandra [29] from the 1st cycle to the 4th cycle (I_d1_–I_d4_) [1]. Density (ρ) and Unit Weight (γ) were measured for natural, dry, and water-saturated samples.

**Table 2:** Number of evaporitic rock blocks, mechanical and physical tests.

Parameters	Test type	Instrument/Formula	Number of rock blocks	Number of tests	Standard
Mechanical	Uniaxial Compressive Strength (UCS) (MPa)	Matest-Cyber Plus Compression Machine (GM19554)	108	260 (C)	ASTM D2938–95 [21]
	Point Load Index (PLI) (MPa)	Matest-Digital Point Load Testers, Set of two spare conical points (A125N, A125–01)	138	357 (C)	ASTM D5731–16 [22]
	Indirect Tensile Strength (ITS) (MPa)	Matest-Digital Point Load Testers, Upper and lower plate with seat ball (A125NA, 125–02)	132	327 (C)	ASTM D3967–08 [23]
	Slake Durability Index (SDI) (%)	Matest-Slake Durability Apparatus (A130)	146	146 (RL)	ASTM D4644–16 [24]
	Schmidt Hardness Value (SHV_RB_) (N)	Proceq Digital Schmidt Rebound Hammer (SH01–009–0081)	137	137 (B)	ASTM D5873–95 [35]
	Sonic Wave Velocity (SWV) (km/s)	Proceq Pundit Lab+ UPV Instrument (CT-133)	115	497 (C)	ASTM D2845–08 [36]
Physical	Natural Density (ρ_n_) (g/cm^3^)	Natural Weight/Volume	144	989 (C)	ISRM Standard-1981 [27]
	Dry Density (ρ_d_) (g/cm^3^)	Dry Weight/Volume	138	355 (C)	ISRM Standard-1981 [27]
	Saturated Density (ρ_s_) (g/cm^3^)	Saturated Weight/Volume	138	355 (C)	ISRM Standard-1981 [27]
	Natural Unit Weight (γ_n_) (kg/m^3^)	ρ_n_ * 9.81 * 102	145	989 (C)	ISRM Standard-1981 [27]
	Dry Unit Weight (γ_d_) (kg/m^3^)	ρ_d_ * 9.81 * 102	138	355 (C)	ISRM Standard-1981 [27]
	Saturated Unit Weight (γ_s_) (kg/m^3^)	ρ_s_ * 9.81 * 102	138	355 (C)	ISRM Standard-1981 [27]
	Water Content (W_c_) (%)	(Total Weight-Dry Weight)/ Dry Weight * 100	149	355 (C)	ISRM Standard-1981 [27]
	Porosity (n) (%)	(Volume of Void/Volume) * 100	138	355 (C)	ISRM Standard-1981 [27]
	Specific Gravity for Cores (G_s_)	Liya-Specific Gravity Test Set (LT-C0066)	144	988 (C)	ASTM C97–02 [28]
	Void Ratio (e)	n/(100- n)	138	355 (C)	ISRM Standard-1981 [27]
Geochemical	X-ray fluorescence (XRF)	PANaLytical X-Ray Diffraction (X'Pert Pro)	29	29	Buhrke et al. [30]

C = Core; RL = Rock Lumps; *B*= Block.

X-ray fluorescence (XRF) elemental analysis was conducted for powder (*<*74 μm) samples (PW 2404 with five analyzing crystals). With an accuracy of 99.99 % and a confidence limit of 96.7 %, the software programs Super Q and Semi Q were used to calculate the concentrations of the tested major elements (oxides in wt.%) [30].

## Limitations

While this dataset provides valuable information on the mechanical, physical, and geochemical properties of evaporitic rocks, its practical application in engineering decisions should carefully consider site-specific variables, project requirements, and environmental considerations such as climate, groundwater conditions, and human activities. Moreover, it focuses on evaporite rocks in Abu Dhabi and its vicinity, which does not reflect the diverse geologic setting of this area; in this context, similar results are crucial for other rock types, particularly carbonate rocks.

## Ethics Statement

The authors have read and follow the ethical requirements for publication in Data in Brief and confirming that the current work does not involve human subjects, animal experiments, or any data collected from social media platforms.

## CRediT authorship contribution statement

**Hasan Arman:** Conceptualization, Data curation, Funding acquisition, Investigation, Methodology, Project administration, Validation, Writing – original draft, Writing – review & editing. **Ahmed Gad:** Conceptualization, Data curation, Investigation, Methodology, Validation, Writing – original draft, Writing – review & editing. **Osman Abdelghany:** Formal analysis, Funding acquisition, Investigation, Methodology, Writing – review & editing. **Bahaa Mahmoud:** Formal analysis, Investigation, Methodology, Writing – review & editing. **Ala Aldahan:** Investigation, Methodology, Writing – review & editing. **Safwan Paramban:** Data curation, Formal analysis, Investigation, Methodology, Validation. **Mahmoud Abu Saima:** Formal analysis, Investigation, Methodology, Writing – review & editing.

## Data Availability

Comprehensive Analysis of Mechanical, Physical, and Geochemical Properties of Evaporitic Rocks in Abu Dhabi, UAE (Original data) (Mendeley Data)
